# Validation of serum C-reactive protein for the diagnosis and monitoring of antibiotic therapy in neonatal sepsis

**DOI:** 10.12669/pjms.336.13927

**Published:** 2017

**Authors:** Ejaz Ahmed, Abdur Rehman, Muhammad Asghar Ali

**Affiliations:** 1 Dr. Ejaz Ahmed (FCPS Pediatrics, FCPS Neonatology Resident), Senior Registrar Neonatology, Children Hospital Complex and the Institute of Child Health, Multan, Pakistan; 2 Dr. Abdur Rehman (FCPS Pediatrics, FCPS Neonatology), Assistant Professor eonatology, Children Hospital Complex and the Institute of Child Health, Multan, Pakistan; 3 Dr. Muhammad Asghar Ali (FCPS Pediatrics), Senior Registrar Neonatology, Children Hospital Complex and the Institute of Child Health, Multan, Pakistan

**Keywords:** Neonatal sepsis, C-reactive protein, Sensitivity, Specificity, Negative predictive value

## Abstract

**Objective:**

To evaluate the adequacy of serum C-reactive protein (CRP) in diagnosing neonatal sepsis and role of CRP in determining the duration of antibiotic treatment in neonatal sepsis.

**Methods:**

In this validation study, we included 135 neonates with suspected diagnosis of sepsis within duration of nine months from September 2016 to May2017 in Children Hospital Multan. Blood samples were drawn from every neonate for culture sensitivity and measurement of serum C-reactive proteins. In all suspected neonates, empirical antibiotics e.g. Gentamycin or Ampicillin were started after taking blood samples. Serum CRP levels >5 mg/dl were marked as positive results. 2^nd^ blood samples for measurement of serum CRP were taken after 72 hours of the first sample. There were two primary endpoints; one to determine the sensitivity and specificity of CRP against blood culture and second was to determine the negative predictive value of CRP in determining the duration of anti-biotic in neonates presenting with sepsis.

**Results:**

Out of these 135 babies, 102 (75.5%) were confirmed to have sepsis using blood culture reports. CRP results were Positive in 85 (62.9%) neonates on first baseline measurement and were positive in 103 (76.29%) neonates after 72 hours of admission. The sensitivity of CRP in diagnosing sepsis was 98.03%, specificity was 91.0%, positive predictive value (PPV) was 97% and negative predictive value (NPV) was 93.7%. The mean duration of antibiotic treatment in CRP guided group was 5.03 days versus 7.02 days in standard treatment duration group (p-value <0.001). The NPV of CRP in determining the duration of antibiotics was 100.0%.

**Conclusion:**

Serum CRP level is a reliable test in establishing the diagnosis of neonatal sepsis. It accurately monitors the duration of antibiotic therapy and results in significant reduction in the treatment duration of neonatal sepsis.

## INTRODUCTION

Neonatal sepsis (NS) is a very challenging scenario for the neonatologists, because most of these neonates present with atypical symptoms and most of these mimic with non-infectious causes, making the exact and timely diagnosis very challenging.^1^,^2^ Any invasive bacterial infection occurring within first month of life is defined as neonatal sepsis, it of early onset if occurs within first week of life and late onset if occurs in after first week of one month.^3^ Prevalence rate of NS has been reported to be 10/1000 to 15/1000 live births in developed world and 15/1000 to 25/1000 live births in South Asia.^1^,^4^ NS is responsible for 30 to 40% of total neonatal mortalities in developing countries.^5^

The current recommendation is to treat septic neonates for 48 to 72 hours if blood culture reports are negative and for 7 to 14 days if culture report is positive.^6^,^7^ According to different studies, about 11% to 23% neonates are treated wrongly for sepsis but they are not having it.^6^-^8^ This not only results in anti-biotic resistance, it also has many other short-term complications (e.g. pain and infection) and some long term complications (e.g. hearing disorder and necrotizing enterocolitis).^9^,^10^

In addition, different organisms causing neonatal spectrum have different spectrum of infection. So instead of following the strict protocols of anti-biotic duration, the anti-biotic duration should also be regionalized according to the causating organism.^11^ Therefore, there is a need to look for rapid diagnostic evaluation tests for NS instead for culture sensitivity reports. Studies have shown that serum C-reactive protein (CRP) levels may help in early diagnosis and in determination of the duration of anti-biotic treatment.^12^,^13^ These C-reactive protein (CRP) are synthesized in liver as a result of insult to foreign agents and remains on peak during inflammatory process and then decrease rapidly after the infection is over (half-life 19 hours).^10^,^14^

In present study, we evaluated the adequacy of serum CRP in diagnosing neonatal sepsis and their role in determining the duration of antibiotic treatment in neonates presenting with suspicion of septicemia.

## METHODS

In this validation study, we included 135 neonates, who presented in neonatology unit of Children Hospital Multan. These patients were selected within duration of 9 months from September 2016 to May 2017. We took approval from IRB board of the hospital before starting the study. All neonates with suspected diagnosis of sepsis were selected. We used following criteria to evaluate neonatal sepsis; presence of unexplained fever or hypothermia, irritability, poor or no feeding, lethargy, respiratory dysfunction (e.g. apnea or tachypnea), cardiovascular dysfunction (e.g. intermittent tachycardia or bradycardia and cold peripheries), presence of maternal risk factors e.g. foul smelling vaginal discharge, rupture of membranes for > 18 hours, history of fever in pre-natal or post-natal period. Neonates having birth weight <1.5 Kg, birth asphyxia and already taking any antibiotic treatment were excluded.

Blood samples were drawn from every neonate for culture sensitivity and measurement of serum C-reactive proteins. In all suspected neonates, empirical antibiotics e.g. Gentamycin and Ampicillin were started after taking blood samples. Serum CRP levels >5 mg/dl were marked as positive results.^15^ Antibiotics if needed were changed after receiving culture sensitivity reports. The 2^nd^ blood sample for measurement of serum CRP were taken after 72 hours of the first sample. Two consecutive positive CRP levels were confirmed as presence of neonatal sepsis. Moreover, two consecutive negative serums CRP were confirmed as absence of neonatal sepsis. Antibiotics were stopped in neonates having negative CRP results and we waited for culture reports of these neonates for further confirmation of septicemia. Blood cultures were followed for five days in CRP guided group and for seven days in standard treatment group and for positive result reports. In neonates, having two consecutive positive CRP levels. These neonates were divided into two groups using simple random sampling (Lottery method); in one group antibiotics were continued, blood samples were taken every day to measure serum levels of CRP, and the antibiotics were stopped as soon as the serum CRP became negative. While in 2^nd^ group, antibiotics were continued for seven days and on 7^th^ days serum CRP were measured to determine positive or negative values. If CRP results were positive, then antibiotics were continued for more duration until the CRP results became negative.

After withdrawal of antibiotics, all these neonates were kept 48 hours (after stopping the anti-biotics therapy) in hospital to monitor any incidence of fever again within this period, if it happens then the plan was start the antibiotic course again.

There were two primary endpoints of this study, one was to determine the sensitivity and specificity of CRP against blood culture to determine the neonatal sepsis and secondary end-point was to determine the negative predictive value of CRP in determining the duration of antibiotics in neonates presenting with sepsis.

For measurement of sensitivity, and specificity we use 2x2 tables. Tables were made using SPSS v23. Mean duration of antibiotics in subgroups was compared using independent sample statistics. Frequencies were calculated for positive and negative serum CRP levels, blood culture reports and relapse rate.

## RESULTS

The mean age of the neonates in this study was 6.7±4.9 days. Out of 135 babies, 77 (57.1%) were male and 58 (42.9%) were female. Most of babies, 86 (63.8%) presented in first week of life, 40 (29.6%) in second week of life and remaining, 9 (6.6%) presented in within one month of life. Out of these 135 babies, 102 (75.5%) were confirmed to have sepsis using blood culture reports. CRP results were Positive in 85 (62.9%) neonates on first baseline measurement and were positive in 103 (76.29%) neonates after 72 hours of admission. The sensitivity of CRP in diagnosing sepsis was 98.03%, specificity was 91.0%, positive predictive value (PPV) was 97% and negative predictive value (NPV) was 93.7%.

Most of the newborns were infected with gram negative organisms 75.5% versus only 24.5% with gram positive infections. Among grams negative organisms, *E-coli, Klebsiella Pneumoniae*, and *Pseudomonas Species* were the most common organisms. Among gram positive organisms, Staphylococcus Aureus and *Staphylococcus Epidermidis* were most common causating agents of neonatal sepsis (Table-I).

**Table-I:** Etiological organisms of sepsis.

*Micro-organisms*		*Frequency (%age)*
Grams Negative Organisms (N=77)	Escherichia Coli	25 (24.5%)
	Klebsiella Pneumoniae	23 (22.5%)
	Pseudomonas Species	18 (17.6%)
	Acinetobacter	9 (8.8%)
	Enterobacter Cloacae	2 (1.9%)
Gram Positive Organisms (N=25)	Staphylococcus Aureus	12 (11.8%)
	Staphylococcus Epidermidis	7 (6.8%)
	α-Hemolytic Streptococci	4 (3.9%)
	Enterococcus Faecalis	2 (1.9%)

Out of 103 neonates who had positive CRP levels on two consecutive readings, 55 neonates received CRP guided antibiotic therapy and the remaining 48 neonates received standard antibiotic duration treatment. In CRP guided group, there were 41 neonates in whom antibiotics were stopped in ≤5 days of therapy and 13 neonates the duration of antibiotics was 6-7 days and in one patient CRP was positive on 7^th^ day and became negative on 9^th^ day. In neonates receiving standard antibiotic treatment, CRP at 7^th^ day was negative in all neonates except one, in whom antibiotics duration was eight days. The mean duration of antibiotic treatment in CRP guided group was 5.03 days versus 7.02 days in standard treatment duration group (p-value <0.001).

There was no relapse case of sepsis in any of the study neonates. The NPV of CRP in determining the duration of antibiotics was 100.0%.

## DISCUSSION

Neonatal sepsis is one of the commonest causes of morbidity and mortality in neonates. Mortality rate is very high if left untreated. Blood culture is the gold standard test for the diagnosis of neonatal sepsis. However, this test is time consuming and is not available in many hospitals of Pakistan so there is a need to look other easily and rapidly available methods for the diagnosis of neonatal sepsis. Among the various hematological factors studied for the early diagnosis of sepsis, CRP is one of them and is most widely studied.

As regards the primary end points of this study, we found 98.03% sensitivity and 91.0% specificity of CRP in confirming the diagnosis of sepsis in neonates after 72 hours of admission. These results are comparable to other conducted studies. Kumar et al.^16^ found 95.2% sensitivity and 85.3% specificity of CRP in neonates with proven sepsis. Nuntnarumit et al.^17^ found 100.0% sensitivity and 94.0% specificity of CRP in neonates with proven sepsis or those having local infections. Benitz et al.^18^ concluded that the sensitivity of CRP in diagnosing neonatal sepsis is increased with the passage of time. They found sensitivity of CRP only 40.0% in first 24 hours of sepsis and 90.0% after 24 hours of infection.

However some studies have found low sensitivity and specificity of CRP in diagnosing the neonatal sepsis. Hisamuddin et al.^15^ found only 76.92% sensitivity and 53.49% specificity of CRP after 72 hours of initial diagnosis in ruling out sepsis. Saeed et al.^19^ also found similar results about CRP sensitivity and specificity. The reasons for these differences are not known.

**Table-II:** Sensitivity and Specificity of serum CRP against Blood Culture.

Sensitivity	98.03
Specificity	91.0%
Positive Predictive Value	97.0%
Negative Predictive Value	93.7%

We also found 100% negative predictive value of CRP in determining the duration of antibiotics. In our study, there was no incidence of relapse in CRP-guided and standard treatment group and duration of antibiotics administration was also shorter 5.03 versus 7.02 days respectively. Siddaiah et al.^20^ also found similar results regarding reduction in the duration of antibiotics. These authors found NPV of CRP 100% in determining the duration of antibiotics. They concluded that antibiotics duration is reduced to five days in 54% CRP-guided neonates. These authors only included low birth weight (LBW) neonates in their study. Coggins et al.^21^ also conducted study in LBW neonates with sepsis and found significant in antibiotics duration using CRP guided therapy. Jaswal et al.^14^ and Ehl et al.^22^ found 100% and 99.0% NPVs of serum CRP levels in determining the duration of antibiotics.

Based on these results and other studies, we can conclude that serum CRP level is a reliable test in establishing the diagnosis of sepsis in neonates and it accurately monitors the duration of antibiotic therapy in these neonates and results in significant reduction in the treatment duration of neonatal sepsis.

### Authors’ Contribution

**EA:** Conceived, designed the study, did data analysis and prepared the manuscript.

**AR and MAA:** Helped in data analysis, did review

**EA:** Takes the responsibility and is accountable for all aspects of the work in ensuring that questions related to the accuracy or integrity of any part of the work are appropriately investigated and resolved.
